# Case in Which Numerous Small Cysts, Some of Which Contained Pus, and Portions of Lymph, Were Found in the Ventricles of the Heart

**Published:** 1826-09

**Authors:** 

**Affiliations:** St. George's Hospital


### DISEASE OF THE HEART.

*Case in which numerous small Cysts, some of which contained Pus, and portions of Lymph, were found in the Ventricles of the Heart.* Treated at St. George's Hospital, by Dr. Young.

July, 1826.—Ann Goodall, ætatis twenty; has been subject for ten years to occasional palpitation of the heart and sensation of choking in the throat, with pain in the left side of the thorax, extending to the back and left shoulder, and accompanied with vomiting. The attack commenced with sense of obstruction at the scrobiculus cordis, rising to the left shoulder, and descending the arm to the veins and little finger of the left hand. At first, these paroxysms occurred very rarely—once only in several months, and did not continue more than a hour or two at each time. Within the last two years, they have become much more frequent. In the intervals, she reports that she was perfectly free from ailment.

Since Christmas last, these attacks have increased in frequency and violence, and she has suffered four weeks, with only one intermission, from the present attack. *Pulse above two hundred*, occasionally too rapid to be counted; with throbbing of the carotid arteries communicated to the veins, through which the rapidity of the circulation is remarkably visible. The catamenia have not appeared for the last seven months. She sleeps only on the right side, and with her head raised. Burning heat is felt over the left side of the chest, with a sensation of great anxiety at the præcordia. Skin cool; no thirst; tongue clean; bowels costive; sleeps ill.

It is worthy of remark, that during one interval of twenty-four hours, which took place while she was in the hospital, the pulse was ninety, and the symptoms greatly abated. With this exception, however, the symptoms continued to increase in violence until a few days before her death, when she became insensible, with occasional screaming and convulsions, particularly of the muscles of the upper extremities. The lower extremities became œdematous, and gangrene began to appear on the feet; and she died, August 9th.

*Dissection.*—The feet and legs were in a state of mortification, with large vesications on the surface, and œdema of the whole subcutaneous texture of both limbs, as high as the pelvis.

The lungs were more solid than usual, and contained a large quantity of watery fluid.

The heart was rather large, and fatter than is generally the case at so early an age. On opening the right ventricle, a quantity of solid substance was perceived, the nature of which it was at first difficult to discover, as it had some of the appearance of coagulated blood; but it was evident that a part of it only was the result of coagulation after death, and the remainder looked more like the contents of an aneurismal sac. On more accurate examination, however, it was found to consist principally of effused lymph, in masses of various shapes and sizes, and in different stages of formation; the colour being for the most part a brownish yellow, but in some parts darker, as if mixed with blood. Many portions were loose and detached, but others adherent to the texture of the heart. Some formed distinct globular cysts, varying from the size of a pea to that of a small walnut; one cyst of the latter size being situated at the bottom of the ventricle. When these were cut open, the inner surface was reticulated, (in the same manner as may often be perceived in abscesses in the liver;) and some of them contained a few drops of pus, while others were entirely empty. The greater part of the lymph, however, was in solid masses, easily torn or broken by pressure. The lymph was most abundant at the lower insertions of the fleshy columns of the tricuspid valve, but extended also into every hollow and sinus formed by the reticulated texture of the sides of the ventricles, in the lower half of its cavity, so as to make it doubtful, on first cutting the substance of the heart, whether the lymph and pus were situated in the cavity of the ventricle, or whether they were in small cells in the muscular texture. But the lymph could every where be raised, so as to show the smooth surface formed by the serous lining of the heart, except in one or two places, where it was doubtful whether there was not a slight abrasion of the membrane; though it seemed, on the whole, more probable that this appearance arose from a firmer adhesion of the lymph, with which a portion of the membrane had been also raised by the handle of the knife.

In the left ventricle, an exactly similar effusion of lymph was discovered, but in smaller quantity.

On making a longitudinal section of one of the pillars of the mitral valve, no distinct muscular appearance could be found, but a yellow substance was seen, in which fibres were only partially evident, between which substance and the serous membrane of the heart a small quantity of blood had recently been effused. In the upper part of the left ventricle, near its junction with the auricle, a considerable portion of the muscle was also partially disorganised, but in a smaller degree; the yellow substance being here in granules, mixed with the fibres, and with spots of effused blood; and a branch of the coronary artery could here be seen nearly obliterated by coagulation of the blood within it. A few smaller portions, of a similar appearance, were also seen in other parts of this as well as of the right ventricle.

The brain was natural in appearance, except in the upper part of the left corpus striatum, which was softened and of a yellow colour to about half its depth; and the circulation in the internal carotid artery of the same side must have been completely obstructed by a firmly adherent coagulum, which was found within it at the side of the sella turcica. The dura mater was much more opake than usual.

